# Asymptomatic Malaria and Associated Risk Factors among School Children in Sanja Town, Northwest Ethiopia

**DOI:** 10.1155/2014/303269

**Published:** 2014-07-07

**Authors:** Ligabaw Worku, Demekech Damtie, Mengistu Endris, Sisay Getie, Mulugeta Aemero

**Affiliations:** ^1^School of Biomedical and Laboratory Sciences, University of Gondar, P.O. Box 196, Gondar, Ethiopia; ^2^Department of Biology, University of Gondar, P.O. Box 196, Gondar, Ethiopia

## Abstract

*Introduction*. Asymptomatic malaria is prevalent in highly endemic areas of Africa and is new challenge for malaria prevention and control strategies. *Objective*. To determine the prevalence of asymptomatic malaria and associated risk factors among school children in Sanja Town, northwest Ethiopia. * Methods.* A cross-sectional study was conducted from February to March 2013, on 385 school children selected using stratified proportionate systematic sampling technique. Pretested questionnaire was used to collect sociodemographic data and associated risk factors. Giemsa-stained thin and thick blood films were examined for detection, identification, and quantification of malaria parasites. Data were entered and analyzed using SPSS 20.0 statistical software. Multivariate logistic regression was done for assessing associated risk factors and proportions for categorical variables were compared using chi-square test. *P* values less than 0.05 were taken as statistically significant. *Results*. The prevalence of asymptomatic malaria was 6.8% (*n* = 26). The majority of parasitemic study participants had low parasite density 65.5% (17/26). Level of grade, age, bed net usage, and frequent exposure to malaria infection were associated with risk of asymptomatic malaria. *Conclusion.* Asymptomatic malaria was low in this study area and is associated with level of grade, age, bed net usage, and frequent exposure to malaria infection.

## 1. Background

Malaria is one of the most widespread human parasitic diseases ranking first in terms of its socioeconomic and public health importance in tropical and subtropical region of the world, especially in sub-Saharan African and Southeast Asian countries [1–4].

Various estimates have been made to measure the global burden of malaria. In 2010, the World Health Organization estimates more than 216 million cases of malaria and 655.000 deaths occur every year worldwide, with 106 countries at risk of malaria infection, among which 91% of deaths occurred in sub-Saharan Africa, 6% in Southeast Asia, and 3% in Eastern Mediterranean Region [1, 5, 6].

Malaria is also major public health problem and obstacle to socioeconomic development in Ethiopia [7]. About 75% of the geographic area of the country has malaria transmission (defined as areas <2,000 m), with 68% (i.e., 54.2 million) of the country's total population living in these areas [8]. According to the Federal Ministry of Health (FMOH) 2009/2010 report, malaria accounts for up to 14% of outpatient consultations (the leading cause of outpatient consultations) and 9% of health facility admissions, with 5–10 million clinical malaria cases each year. However, of these, only one million are reported at the national level, with 462,623 (55.84%) examined and 256,487 (23.68%) confirmed positive by a diagnostic test. According to FMOH reports, approximately 70,000 people die of malaria each year in Ethiopia [9].

The major clinical features of malaria include severe anemia, cerebral malaria, hypoxia, and placental infection during pregnancy [10]. Whereas severe headache, fever, vomiting, anemia, and loss of appetite are the clinical features of uncomplicated malaria; among these, severe anemia and cerebral malaria constitute the major cause of death [11]. In addition, continuous exposures to* Plasmodium *parasites lead to the development of partial immunity for protection against further complication and, consequently, the creation of asymptomatic carrier [12]. Asymptomatic cases provide a fundamental reservoir of parasites and they might become gametocyte carriers, contributing to the persistence of malaria transmission [13]. Also, there are reports that parasites from asymptomatic carrier are more infectious than symptomatic one [14].

Most of the studies of malaria depend on clinical manifestations, severity, and complication because it is the principal cause of malaria-related deaths. Researchers and clinicians have established diagnostic criteria based on the clinical manifestations upon disease onset, which have aided in forming an integrated approach to improving the management and treatment of severe malaria [15].

Diagnosing asymptomatic malaria is not straightforward due to the obvious lack of clinical manifestations and often low level of parasites [16]. Asymptomatic malaria is prevalent in malaria endemic regions and has become a serious cause for concern as efforts are increasing towards eliminating the parasite [17]. Particularly, subpatent malaria is still transmissible and will complicate elimination of malaria in high transmission regions.

In Ethiopia, the current malaria control methods largely focus on early detection of parasite on suspected individual and treatment, insecticide-treated bed nets (ITN) and indoor residual spraying. Since individuals with asymptomatic parasitaemia will not be identified by early detection and treatment programs, they may continue to serve as a source of infection for vector mosquitoes, complicating control measures [14].

Asymptomatic malaria is a new challenge for national strategic plan for malaria prevention and control (2011–2015): a situation in which a human* Plasmodium *reservoir is maintained, with individuals who are not treated because they are not diagnosed, since they are asymptomatic. On the other hand, the diagnosing of such cases becomes difficult because of the low level of parasitemia; therefore, the present study was aimed at determining the magnitude of asymptomatic malaria and associated risk factors among school children of Sanja General Elementary School in Sanja Town, northwest Ethiopia.

## 2. Methods

### 2.1. Study Design

A cross-sectional study was conducted from February 1 to March 30, 2013, at Sanja General Elementary School children aged 6 to 15 years in Sanja Town, northwest Ethiopia.

### 2.2. Study Area and Study Population

The study was carried out in Sanja General Elementary School. The town Sanja is located 792 km far from the capital city Addis Ababa on the roadside to Gondar-Humera. Sanja has an altitude of 1800 m above sea level, with annual rainfall range from 800 to 1800 mm and annual temperature range from 25°C to 42°C. There are two elementary, one junior, and one high school. One health center gives service for the dwellers of the town and the surrounding areas. There is a river known as Sanja that flows throughout the year. Sanja General Elementary School is located on the west of the main road. A total of 2079 (872 male, 1207 female) students were enrolled in the school for 2012/13 academic years. Those students, enrolled in the school during the study period, were used as study population.

### 2.3. Sampling and Sample Size Determination

The sample size was determined using statistical formula (*n* = (*z* ^2^ *p*(1 − *p*)*d* ^2^)) considering 95% confidence interval and 50% prevalence. Based on this assumption 385 study participants were selected from 2079 students. To select the study subjects, the students were first stratified according to their educational level (Grade 1 to Grade 8). Allocation of student was proportional to the number of students in each grade. Finally, the study subjects were selected using systematic sampling by using class roster as the sampling frame. Every fifth study subject was selected for the study.

## 3. Data Collection

### 3.1. Sociodemography and Risk Factors Assessments

Well structured questionnaire based on the known risk factors for asymptomatic malaria was locally developed specifically in English version by the research team. It was later translated into the Amharic, local language of the study area. Comparisons were made on the consistency of the two versions. The questionnaires address students' sociodemographic information and risk factors around. A pretest was conducted and any ambiguous questions and repetitive ideas were corrected. Additional response categories were also added based on the pretest findings.

### 3.2. Detection, Identification, and Quantification of Malaria

It was done according to a well accepted reference (such as Cheesbrough M (200) parasitological tests in district laboratory practice in tropical countries, part 2) [18].

## 4. Operational Definition

### 4.1. Asymptomatic Malaria

A person with no recent history of symptoms and/or signs of malaria who shows laboratory confirmation of parasitemia [19].

### 4.2. Quality Control

To ensure good and reliable result, the following care was taken. Quality control of microscopy involves ensuring that good-quality Giemsa stain is used and staining procedures adhere to recognized methods. In addition, ten percent of the total slide was randomly selected and reexamined at the end by experienced laboratory technologist, who is blind to the first examination result. Double entry of data was done to maintain quality of the data, and before starting analysis, data was checked for completeness and being filled out clearly.

### 4.3. Data Analysis and Interpretation

Data were entered and analyzed using SPSS 20.0 (SPSS Inc., Chicago, 2011) software. Descriptive statistics was used to give a clear picture of background variables. The frequency distribution of both dependent and independent variables was worked out. Multivariate logistic regression was done for assessing associated risk factors and proportions for categorical variables were compared using chi-square test. *P* values less than 0.05 were taken as statistically significant.

### 4.4. Ethical Consideration

Ethical clearance was obtained from research and ethics review committee of School of Biomedical and Laboratory Sciences, University of Gondar. Before starting the actual data collection, permission was obtained from school director. Additionally, after explaining the importance of the study, an informed written consent was obtained from study participant's parent/guardian. An assent was also taken from individual school children. Those students who were positive for malaria infection were treated according to the national protocol.

## 5. Results

### 5.1. Sociodemographic Characteristics

A total of 385 individuals were included in the study. Their mean age was 12.7 years (range 6 to 15 years) with standard deviation of 2.3. Among these, 132/385 (34.3%) were males and the rest 253/385 (65.7%) were females. Of the total 385 school children, 312 (81%) were in the age range of 11–15 years (Tables 1 and 2).

### 5.2. Prevalence of Asymptomatic Malaria

Out of the total school children, 26 (6.8%) were positive for* Plasmodium* species and two* Plasmodium* species,* P. falciparum *and* P. vivax*, were identified with the prevalence of 20/385 (5.2%) and 6/385 (1.6%), respectively. Of the 385 school children, 13/132 (9.8%) and 13/253 (5.1%) of males and females had asymptomatic malaria, respectively. The distribution of asymptomatic malaria among each age group showed that 11/73% (15.1%) of 6–10 years and 15/312 (4.8%) of the 11–15 years were infected. Age significantly associated with both* P. falciparum* and* P. vivax* (Tables 1 and 2).

Regarding the level of parasitemia with respect to age and sex, 17/26 (65.4%) had less than 500 parasites/*μ*L; there was a significant difference in the level of parasitemia among age groups (*χ* ^2^ = 10.1, *P* = 0.006), but there was no significant difference between the sexes (Table 3).

### 5.3. Risk Factors of Asymptomatic Malaria

Risk factors assessment in general showed that level of grade, age, bed net usage, and frequent exposure to malaria infection were associated with risk of asymptomatic malaria. Students who did not have a habit of frequently used bed net were 2.1 times (AOR = 2.1, 95% CI: 1.20, 3.60) more likely to develop asymptomatic malaria. Similarly there was significant difference between exposure rates to malaria infection; more exposed group was 2.12 times (AOR = 2.12, 95% CI: 1.06, 4.40) more likely to have asymptomatic malaria than those who were less exposed. Age and grade significantly associated with asymptomatic malaria with the risk of 1.3 times higher in lower aged and graded groups, while sex, residence, mother/guardian education status, years of stay in the study area and surroundings, previous illness of malaria, and treatment for symptomatic malaria were not associated with asymptomatic malaria (*P* > 0.05) (Table 4).

## 6. Discussion

Malaria in areas of unstable transmission usually follows seasonal patterns of transmission as mosquito populations fluctuate, with the prevalence of parasitaemia at a minimum in the cooler dry season. In this time asymptomatic infections of malaria are critical, as this reservoir is likely responsible for sustaining the parasite population from one transmission season to the next [20]. Besides, asymptomatic malaria cases are difficult due to low parasitic density and availabilities of simple diagnostic methods; asymptomatic carriers especially adults are common in endemic areas and, as potential gametocyte carriers, represent an important reservoir for malaria transmission [21]. Many of these individuals can carry microscopically detectable levels of* Plasmodium* asymptomatically and submicroscopic asymptomatic infections below the limit for microscopic detection that can only be detected using molecular techniques [13].

High prevalence of asymptomatic* Plasmodium* infection was observed 26/385 (6.8%) in this geographical setting; this might be the effect of climate change on highland malaria transmission which is strongly dependent on the temperature, because temperature is known to influence transmission intensity through its effects on the population growth of the mosquito vector and on pathogen development within the vector [22]. This finding is very low compared to a study conducted on school children in Southwest Cameroon, which reported a prevalence of 44.3%, in Yemen 12.8%, in Mount Cameroon region 87.8%, in Osogbo (Nigeria) 25.6%, and in Tanzania 14% [23–27]. The reasons behind this fact remain to be clarified, but we can speculate that this might be associated with biological variability, such as genetic susceptibility related to the presence of sickle cell trait, beta-thalassaemia, or Duffy blood factor [28, 29] and differences in exposure to mosquito bites which are related to the living standard of the community, burden of mosquito vector, type of occupational activity, housing characteristics (e.g., screening, roof materials, and open eaves) [29, 30], and the use of selected preventative measures [31]. In addition, the biology of vector and parasites play an important role in malaria transmission to the extent that it can increase human exposure and limit the selection of control interventions—for example, an outdoor biting pattern limits the use of IRS and bed nets [32]. The diagnostic method/design we used also differs from others.

Among malaria positive study subjects, the prevalence of* P. falciparum *was 76.9%; this is almost similar to conducted in Indonesia 80.2% [33] and but lower than Yemen 96.7% [26]. However, it is very high compared to a study in Southwest Cameroon 17.5% [23] and Kenya 2.9% [29]. On the other hand, the prevalence of* P. vivax* in this study was 23.1%, which is more or less comparable to 19.8% prevalence reported in Indonesia [23]. The sex distribution of asymptomatic malaria in this study showed that 9.8% were males and 5.1% were females. It is very low compared with study conducted in Cameroon, which reported that the prevalence of males was 48.1% and that of females was 41.4% [23]. This prevalence and gender difference might be due to sampled in different season and socioeconomic level that are supported with earlier access to prevention and treatment and presence or absence of mosquito breeding site throughout a year.

Regarding the level of parasitemia, result from this study showed that the majority of parasitemic study participants had low parasite density. This may be due to the low diagnostic sensitivity of routine microscopic examination. The level of parasitemia has significant association with older aged group; this might be due to repeated exposure of malaria infection.

In this study, we had also evaluated the risk factor associated with asymptomatic malaria. An association was observed between asymptomatic malaria and the age of the children where the prevalence decreased from 15.1% among children aged 6–10 years to 4.8% among children aged 11–15 years (*P* < 0.05). Similar result was also reported in native Amazonian populations [34]. This may be because of increased consciousness about the transmission and information on the prevention mechanism of infection. In addition, the intensity of parasite decreases as the level of school grade increases. The results from this study demonstrated that bed net usage frequency and repeated episodes for malaria infection were strongly associated with asymptomatic malaria (*P* < 0.05). This finding is comparable with the study done in Burkina Faso [13].

## 7. Conclusion

Six point eight percent of the school children had asymptomatic malaria. Level of grade, age, bed net usage, and frequent exposure to malaria infection were associated with risk factors identified in this study. Therefore, this indicated that there is a need of further evaluation of the burden of asymptomatic malaria using more sensitive method (PCR) considering different age groups and malaria transmission level to scale up the elimination and eradication program of malaria in the country [32].

## Figures and Tables

**Table 1:** Prevalence of asymptomatic malaria by sex among school children.

Parasite	Number (%) infected				
	Male	Female	Total	*P* value	*χ* ^2^
	*N* = 132	*N* = 253	*N* = 385		
*P. falciparum *	10 (7.6)∗	10 (54)	20 (5.2)	0.128	2.31
*P. vivax *	3 (2.3)	3 (1.2)	6 (1.6)	0.414	0.67
Total	**13 (9.8)**	**13 (5.1)**	**26 (6.8)**	**0.080**	**3.06**

^*^Figures in parentheses indicate percentages.

**Table 2:** Prevalence of asymptomatic malaria by age groups among school children.

Infection	Number (%) infected				
	6–10 yrs	11–15 yrs	Total	*P* value	*χ* ^2^
	*N* = 73	*N* = 312	*N* = 385		
*P. falciparum *	8 (11)∗	12 (3.8)	20 (5.2)	0.014	6.08
*P. vivax *	3 (4.1)	3 (1.0)	6 (1.6)	0.051	3.82
Total	**11 (15.1)**	**15 (4.8)**	**26 (6.8)**	**0.002**	**9.89**

^*^Figures in parentheses indicate percentages.

**Table 3:** Levels of parasitemia by sex and age group among the parasitemic school children.

Parameter	Parasite density distribution per microliter of blood					
	<5000	500–999	≥1,000	Total	*P* value	*χ* ^2^
Sex						
Male	7 (26.9)∗	5 (19.2)	0 (0.0)	12 (46.2)	0.16	3.68
Female	10 (38.5)	2 (7.7)	2 (7.7)	14 (53.8)		
Total	**17 (65.4)**	**7 (26.9)**	**2 (7.7)**	**26 (100)**		
Age/years						
6–10	11 (42.3)	0 (0.0)	0 (0.0)	11 (42.3)	0.006	10.1
11–15	6 (23.1)	7 (26.9)	2 (7.7)	15 (57.7)		
Total	**17 (65.4)**	**7 (26.9)**	**2 (7.7)**	**26 (100)**		

^*^Figures in parentheses indicate percentages.

**Table 4:** Multivariate analysis of factors potentially associated with asymptomatic malaria infection among school children.

Risk factors	Malaria		Total	OR (95%CI)		*P* value
	Positive	Negative		COR	AOR	
Sex						
Male	13 (9.8)∗	119 (90.2)	132 (34.3)	2.02 (0.85, 4.80)	0.966 (0.344, 2.713)	0.125
Female	13 (5.1)	240 (94.9)	253 (65.7)	1.00	1.00	
Age (years)						
6–10	11 (15.1)	62 (84.9)	73 (19)	3.51 (1.43, 8.59)	1.255 (1.049, 1.331)	0.015
11–15	15 (4.8)	297 (95.2)	312 (81)	1.00	1.00	
Resident						
Urban	19 (6.6)	269 (93.4)	288 (74.8)	1.00	1.00	0.840
Rural	7 (7.2)	90 (92.8)	97 (25.2)	1.10 (0.41, 2.89)	1.278 (0.428, 3.830)	
Grade						
One–four	17 (13.8)	111 (86.2)	128 (33.2)	4.22 (1.71, 10.60)	1.285 (1.007, 1.053)	0.040
Five–eight	9 (3.5)	248 (96.5)	257 (66.8)	1.00	1.00	
Mothers/guardian educational status						
Illiterate	5 (3.5)	139 (96.5)	144 (37.4)	0.02 (0.08, 1.04)	3.767 (0.933, 15.206)	1.107
Reading and writing/1 year	13 (7.7)	159 (92.3)	172 (44.6)	0.66 (0.24, 1.85)	1.452 (0.460, 4.581)	
2 years at school and above	8 (11)	65 (89)	73 (19.0)	1.00	1.00	
Family size						
≤4	10 (9)	101 (91)	111 (28.8)	1.00	1.00	0.562
5–7	12 (5.6)	203 (94.4)	215 (55.8)	0.60 (0.60, 1.55)	2.304 (0.776, 6.843)	
≥8	4 (6.8)	55 (93.2)	59 (15.4)	0.73 (0.18, 2.71)	3.138 (0.630, 15.588)	
Years of stay						
<5 years	3 (4.5)	66 (95.5)	69 (17.9)	1.00	1.00	0.218
5–10 years	10 (10.4)	84 (89.6)	94 (24.4)	2.54 (0.61, 12.18)	1.018 (0.178, 5.832)	
>10 years	13 (5.9)	209 (94.1)	222 (57.7)	1.33 (0.34, 6.06)	1.144 (0.240, 5.177)	
Swampy area						
Present	2 (3.7)	52 (96.3)	54 (14.0)	0.49 (0.08, 2.24)	1.189 (0.212, 6.004)	0.339
Absent	24 (7.3)	307 (92.7)	331 (86.0)	1.00		
Bed net usage						
Always	2 (1.7)	115 (98.3)	117 (30.4)	1.00	1.00	0.011
Sometimes/not at all	24 (9)	244 (91.0)	268 (69.6)	5.66 (1.27, 35.23)	2.111 (1.200, 3.604)	
Ever sick by malaria before						
Yes	23 (8.0)	264 (92.0)	284 (73.7)	2.97 (0.76, 11.82)	0.156 (0.002, 1.574)	0.150
No	3 (3.1)	95 (96.9)	101 (26.3)	100	1.00	
Frequency of illness						
<5 malaria episodes	15 (4.5)	321 (95.5)	336 (87.3)	1.00	1.00	0.001
>5 malaria episodes	9 (14.9)	40 (85.1)	49 (12.7)	4.82 (1.81, 12.67)	3.185 (1.063, 7.064)	
Treated for symptomatic malaria cases						
Yes	23 (7.8)^◊^	243 (92.2)	266 (69.1)	1.00	1.00	0.076
No	3 (3.3)^◊^	107 (96.7)	110 (30.9)	3.38 (0.94, 14.44)	5.132 (0.596, 44.165)	
Total	**26 (6.8)**	**359 (93.2)**	**385 (100)**			

^*^Figures in parentheses indicate percentages.

^◊^Only for previous malaria episode.
